# Elevating understanding: Linking high-altitude hypoxia to brain aging through EEG functional connectivity and spectral analyses

**DOI:** 10.1162/netn_a_00352

**Published:** 2024-04-01

**Authors:** Carlos Coronel-Oliveros, Vicente Medel, Grace Alma Whitaker, Aland Astudillo, David Gallagher, Lucía Z-Rivera, Pavel Prado, Wael El-Deredy, Patricio Orio, Alejandro Weinstein

**Affiliations:** Latin American Brain Health Institute (BrainLat), Universidad Adolfo Ibáñez, Santiago, Chile; Global Brain Health Institute (GBHI), University of California, San Francisco (UCSF), San Francisco, CA, USA and Trinity College Dublin, Dublin, Ireland; Centro Interdisciplinario de Neurociencia de Valparaíso (CINV), Universidad de Valparaíso, Valparaíso, Chile; Brain and Mind Centre, The University of Sydney, Sydney, Australia; Department of Neuroscience, Universidad de Chile, Santiago, Chile; Advanced Center for Electrical and Electronics Engineering (AC3E), Federico Santa María Technical University, Valparaíso, Chile; Chair of Acoustics and Haptics, Technische Universität Dresden, Dresden, Germany; Centro de Investigación y Desarrollo en Ingeniería en Salud, Universidad de Valparaíso, Valparaíso, Chile; NICM Health Research Institute, Western Sydney University, Penrith, New South Wales, Australia; School of Psychology, Liverpool John Moores University, Liverpool, England; Escuela de Fonoaudiología, Facultad de Odontología y Ciencias de la Rehabilitación, Universidad San Sebastián, Santiago, Chile; Instituto de Neurociencia, Facultad de Ciencias, Universidad de Valparaíso, Valparaíso, Chile

**Keywords:** Oxygen supply, High-altitude hypoxia, Aging, EEG, Power spectrum, 1/f aperiodic activity, Functional connectivity

## Abstract

High-altitude hypoxia triggers brain function changes reminiscent of those in healthy aging and Alzheimer’s disease, compromising cognition and executive functions. Our study sought to validate high-altitude hypoxia as a model for assessing brain activity disruptions akin to aging. We collected EEG data from 16 healthy volunteers during acute high-altitude hypoxia (at 4,000 masl) and at sea level, focusing on relative changes in power and aperiodic slope of the EEG spectrum due to hypoxia. Additionally, we examined functional connectivity using wPLI, and functional segregation and integration using graph theory tools. High altitude led to slower brain oscillations, that is, increased *δ* and reduced *α* power, and flattened the 1/f aperiodic slope, indicating higher electrophysiological noise, akin to healthy aging. Notably, functional integration strengthened in the *θ* band, exhibiting unique topographical patterns at the subnetwork level, including increased frontocentral and reduced occipitoparietal integration. Moreover, we discovered significant correlations between subjects’ age, 1/f slope, *θ* band integration, and observed robust effects of hypoxia after adjusting for age. Our findings shed light on how reduced oxygen levels at high altitudes influence brain activity patterns resembling those in neurodegenerative disorders and aging, making high-altitude hypoxia a promising model for comprehending the brain in health and disease.

## Author Summary

Exposure to high-altitude hypoxia, with reduced oxygen levels, can replicate brain function changes akin to aging and Alzheimer’s disease. In our work, we propose high-altitude hypoxia as a possible reversible model of human brain aging. We gathered EEG data at high altitude and sea level, investigating the impact of hypoxia on brainwave patterns and connectivity. Our findings revealed that high-altitude exposure led to slower and noisier brain oscillations and produced altered brain connectivity, resembling some remarkable changes seen in the aging process. Intriguingly, these changes were linked to age, even when hypoxia’s effects were considered. Our research unveils how high-altitude conditions emulate brain patterns associated with aging and neurodegenerative conditions, providing valuable insights into the understanding of both normal and impaired brain function.

## INTRODUCTION

It has been estimated that around 81.6 million people live at >2,500 masl (1.07% of the world’s population) (Tremblay & Ainslie, 2021). In addition, many people work at high altitudes, such as mining workers, who may work at >2,000 masl (Siliverstovs & Herzer, 2007). Working at high altitude is considered a condition of risk for developing health problems (Virués-Ortega et al., 2004), reducing productivity and increasing the probability of accidents (Chiu et al., 2014). Some symptoms associated with hypoxia at high altitudes may transiently persist after returning to sea level. Therefore, the negative outcomes related to acute and chronic hypoxia are of relevance to public health (Hu et al., 2017). The search for biomarkers of health impairments constitutes an avenue for developing treatments, therapies, and devices to treat the health and cognitive problems related to high altitude.

Acute exposure to changes in oxygen—such as when quickly blowing up balloons or climbing high mountains without preparation—can cause a series of neurological symptoms that resemble known hallmarks of aging and neurodegenerative diseases, including changes in the autonomic system, cognitive control, memory, and executive functions (Asmaro et al., 2013; de Aquino Lemos et al., 2012; Malle et al., 2016; Ochi et al., 2018). An example of this state is high-altitude hypoxia, understood as the arterial partial pressure of oxygen (PaO2) triggered by the decline of air oxygen pressure due to altitude, which occurs when subjects are acutely exposed to an altitude above 2,500 masl without preparation (Virués-Ortega et al., 2004). Interestingly, the severity of these symptoms can increase depending on prior acclimatization and the physical activity undertaken by the subject, which suggests that acutely perturbing oxygen supply may generate brain dysfunction due to the brain’s slow adaptation to the increase in energetic demands (Petrassi et al., 2012).

The reversible functional brain alterations triggered by high-altitude hypoxia may be used to understand the functional disturbances associated with healthy aging. Brain function slowly adapts to the availability of energetic supply, which is affected by internal processes such as metabolic aging, as well as by external resources such as oxygen availability. Although brain aging has been thought to affect various functional measures of brain activity, delineating the energetic component from the different sources, for example, neurodegeneration, that occur during aging is essential to the further understanding of healthy and pathological aging.

Hypoxia can disrupt different measures of brain function, observed at different spatial and temporal scales. Evidence from electroencephalography (EEG) has shown an increase in low-frequency oscillations accompanied by a decrease in overall EEG power (Babiloni et al., 2014), which has been confirmed under both artificial hypoxia via the use of a hypobaric chamber as well as altitude-induced hypoxia (Babiloni et al., 2014; Ozaki et al., 1995; Zhao et al., 2016), with recovery of the EEG measures related to chronic acclimatization (Zhao et al., 2016).

The perturbation induced by hypoxia may trigger a cascade of metabolic consequences beyond that which can be characterized by local oscillatory power. Indeed, brain signals change in relation to their state, affecting how information is processed and distributed throughout the brain. Background signals can be addressed by the 1/f slope of the spectrum—a measure of the aperiodicity of the signal (Gao et al., 2017; Lendner et al., 2020; Medel et al., 2023; Trakoshis et al., 2020)—while the regional interactions can be characterized by functional connectivity (FC), which attempts to capture shared functional properties (statistical interdependencies) in the brain. Although the exact mechanism underlying these network-state measures remains unclear, strong evidence has shown that aperiodicity (Martínez-Cañada et al., 2023; Voytek et al., 2015; Vyšata et al., 2014) and FC (Gatica et al., 2021, 2022; King et al., 2018; Song et al., 2014) are strongly affected by aging and neurodegenerative diseases (e.g., Alzheimer’s disease).

The present study aims to evaluate the utility of high-altitude hypoxia as a model for investigating disrupted brain function similar to that observed in brain aging and neurodegenerative conditions (Babiloni et al., 2014; King et al., 2018; Martínez-Cañada et al., 2023; Ozaki et al., 1995; Song et al., 2014; Voytek et al., 2015; Vyšata et al., 2014; Zhao et al., 2016). We assessed brain function from measures including oscillatory power, aperiodicity (absence of regular patterns in the signal), and FC in resting-state EEG (RS-EEG) of 16 healthy, low-altitude inhabitants recorded at both sea level and 4,000 masl (acute high-altitude hypoxia). We hypothesize that reduced oxygen levels at high altitudes cause functional changes similar to those found in both aging and Alzheimer’s disease and that this can provide valuable insight with respect to treatment and mitigation of associated deficits.

## METHODS

### Study Design and Ethic Statement

Sixteen healthy volunteers were recruited for the experiment. This study was a collaboration between the University of Valparaíso (Chile), the Universidad Técnica Federico Santa María (Chile), and CODELCO-Andina, within the framework of the ANID-FONDEF ID16I10322 project (Chile). The age of the participants ranged from 19 to 45 years (34 ± 7.9 years). Exclusion criteria included nicotine consumption (smoking), diabetes, and any cardiovascular, pulmonary, or neurological condition (including epilepsy, depression, schizophrenia, or bipolar disorder). Conditions were counterbalanced. Measurements were first obtained from 10 subjects at Codelco-Andina (4,000 masl), and then at the Engineering Faculty of the University of Valparaíso (sea level). For the remaining six subjects, recordings were acquired first at sea level and then at Cristo Redentor (4,000 masl). The study was approved by The University Research Ethics Committee of The University of Manchester, UK (ref. 14374), and was conducted according to the Declaration of Helsinki.

### Data Acquisition and Preprocessing

The measurements were taken between 1–3 hours after arriving at high altitude from sea level. In general, when arriving from sea level, proper adjustment to 4,000 masl takes several days, and requires adequate sleep (Muza et al., 2010). We recorded 10 min (10.3 ± 0.76 min) of eyes-open resting-state EEG (RS-EEG) from 16 healthy participants (Biosemi Active II, 64 channels, sampling rate of 4096 Hz). In addition, four external electrodes were used to record blinking and ocular movements. RS-EEG data was cleaned in MATLAB (MathWorks Natick, MA) using the EEGLab toolbox (Delorme & Makeig, 2004) and custom routines. Visual inspection was performed to identify and remove channels or data blocks containing large artifacts (noisy EEG segments). Recordings were downsampled to 2048 Hz and band-pass filtered between 0.01 and 120 Hz in two stages of FIR filtering. Independent component analysis was used to decompose the data (JADER algorithm, 30 principal components). Components resembling stereotyped artifacts, such as blinks, eye movement, cardiac signal, and muscle activity, were identified and rejected based on their temporal profile, spectral signature, and topography. Then, the signal was reconstructed with the remaining components. Re-reference was performed using the average across electrodes.

### Power Spectral Density, Band Separation and 1/f Analyses

Power spectral density (PSD) was estimated for each channel using the Welch method: Hanning windows of 4 seconds in length (allowing a resolution of 0.25 Hz) and 50% overlap (Welch, 1967). The absolute power within each band was calculated by integrating the PSD functions in the frequency range of interest. We reported the relative power with respect to the broadband spectrum (0.5–30 Hz).

The aperiodic 1/f component of the EEG signals was analyzed using the FOOOF algorithm, as described by Donoghue et al. (2020). FOOOF is an open-source Python package that estimates the aperiodic components of neural power spectra using a combination of Gaussian fitting and background frequency scaling. Thus, it separates oscillatory from background aperiodic activity. The FOOOF analysis was performed on the PSD for each participant. We accounted for aperiodic activity in the frequency range of 4–40 Hz. From the model, we here report the spectral exponent.

Finally, for FC analysis, signals were band-pass filtered with a Bessel 3rd order filter in the common EEG frequency bands *δ* (0.5–4 Hz), *θ* (4–8 Hz), *α* (8–13 Hz), and *β* (13–30 Hz).

### Functional Connectivity

The weighted phase lag index (wPLI), an extension of the PLI, was used as a measure of FC in the sensor space (between channels) (Kida et al., 2016; Vinck et al., 2011) for the filtered RS-EEG signals. The wPLI was used to avoid the problem of overestimating FC by volumetric conduction (Kida et al., 2016; Vinck et al., 2011). The metric quantifies frequency synchronization, but also weights the phase lags and leads by the imaginary component of the cross-spectrum, reducing the sensitivity of the metric to noise and increasing its power to detect true changes in FC (Kida et al., 2016). Furthermore, previous studies showed that network topology estimated from phase-phase interactions, that is, those obtained through wPLI, were robust, stable, and consistent (Chu et al., 2012), and no real differences were observed in FC analysis using phase-phase and amplitude-amplitude interactions (Mostame & Sadaghiani, 2020). Specifically, wPLI uses the sign of the imaginary part of the cross-spectrum between two signals *x* and *y*, denoted by *PSD*_*xy*_, weighted by the imaginary part of its magnitude

$$\text{wPLI}=\frac{\left|E\left(\left|\text{Imag}\left(PSD_{xy}\right)\right|\right)sgn\left(\text{Imag}\left(PSD_{xy}\right)\right)\right|}{E\left(\left|\text{Imag}(PSD_{xy}\right|\right)}$$

where the *Imag* function returns the imaginary part of the argument, *sgn* the sign (1 for positive values or −1 for negative ones), and *E* the expected value (computed as the mean). The wPLI was calculated using the MNE package for Python (Gramfort et al., 2013) based on the equations of Vinck et al. (2011), using the multitaper method, and segmenting the signals in 12-second epochs (Fraschini et al., 2016). FC matrices were calculated within each epoch, and the grand-averaged functional connectivity matrix across epochs was finally used for further analysis and quantifications. We built 64 × 64 nodes (channels) undirected and weighted (bounded between 0 and 1) FC matrices.

### Graph Analysis

Starting from the FC matrices, we first applied a proportional threshold to remove spurious connectivity values (van den Heuvel et al., 2017). FC matrices were subsequently binarized and graph measures were computed. We employed global efficiency and transitivity, which are metrics related to integration and segregation, respectively (Rubinov & Sporns, 2010).

Global efficiency is based on paths and was defined as (Latora & Marchiori, 2001)

$$E=\frac{1}{n}\sum_{i}^{n}E_{i}=\frac{1}{n}\sum_{i}^{n}\frac{\sum_{j\neq i}^{n}d_{ij}^{-1}}{n-1}$$

with *E*_*i*_ as the nodal efficiency, *n* = 64 the total number of nodes, and *d*_*ij*_ the shortest path that connects two nodes *ij*. Global efficiency is bounded between 0 and 1. Higher values are expected to be found in very integrated networks, where nodes can easily reach each other; values near 0 means the opposite.

Transitivity is related to the count network’s triangular motifs, and it was computed as (Newman, 2003)

$$T=\frac{\sum_{i}^{n}2t_{i}}{\sum_{i}^{n}k_{i}\left(k_{i}-1\right)}$$

where *t*_*i*_ corresponds to the number of triangles around the node *i*, and *k*_*i*_ the node degree. The first one is defined as

$$t_{i}=\frac{1}{2}\sum_{j\neq i}^{n}\sum_{h\neq j,h\neq i}^{n}a_{ij}a_{ih}a_{jh}$$

with *a*_*ij*_ = 1 if two nodes *ij* are connected in the graph, and 0 otherwise. On the other hand, node degree is calculated as

$$k_{i}=\sum_{j}^{N}a_{ij}$$

and, in the case of weighted networks, *a*_*ij*_ captures both the existence of connectivity and its strength, taking a value between 0 and 1. Transitivity captures the degree of local (short-range) interactions between nodes and, in more common words, it counts “how many of my friends are also friends between them.” Higher values are expected from segregated networks, and 0 is the opposite (e.g., completely random networks). At the more local level, we calculated the nodal clustering coefficient defined as (Watts & Strogatz, 1998)

$$C_{i}=\frac{1}{n}\sum_{i}^{N}\frac{2t_{i}}{k_{i}\left(k_{i}-1\right)}$$

The main difference between the transitivity and clustering coefficient relies on the normalization performed: clustering is normalized individually for each node, while transitivity is normalized collectively. Using transitivity avoids the problem of extorting the metric by nodes with low degrees (Rubinov & Sporns, 2010).

To avoid the arbitrariness of choosing a single proportional threshold, we used a range of thresholds from 5% to 40% with linear increments of 1%. Proportional thresholding was used to ensure that all networks have the same number of connections across different thresholds, considering that graph metrics are very sensitive to network density (van den Heuvel et al., 2017). FC matrices were then binarized after thresholding, to divorce possible alterations of EEG network topology from overall FC (the average strength of FC) triggered by altitude (van den Heuvel et al., 2017). We reported the area under the curve (AUC) of each graph metric as a function of the threshold (Ginestet et al., 2011).

### Statistical Analysis

All group comparisons, such as relative power, overall FC, and network metrics, were performed with nonparametric permutation tests. These tests are suitable when using small sample sizes, and do not require any assumptions regarding normality (Nichols & Holmes, 2002). The real difference between groups (computed as the mean difference) was compared with the distribution obtained from 10,000 random surrogates; they were acquired by randomly reassigning the measures between groups (sea level and altitude). Results were considered statistically significant for *p* values < 0.05. In addition to the permutation tests, we reported the Cohen’s D effect size. Correlations between age and EEG measurements were computed with Pearson’s correlation. To analyze the possible confounding effects of age on the computed metrics, we repeated the principal analysis of each subsection, but first partialling out the effect of age from measurements. We compared both conditions, that is, sea level and altitude, using the residuals from the regression with age. All *p* values were corrected for multiple comparisons with the Benjamini-Hochberg method (Benjamini & Hochberg, 1995), to decrease the probability of making a type I error (false positives).

## RESULTS

### High-Altitude Hypoxia Increases the Relative Power of Slow Oscillations

**Figure 1.**
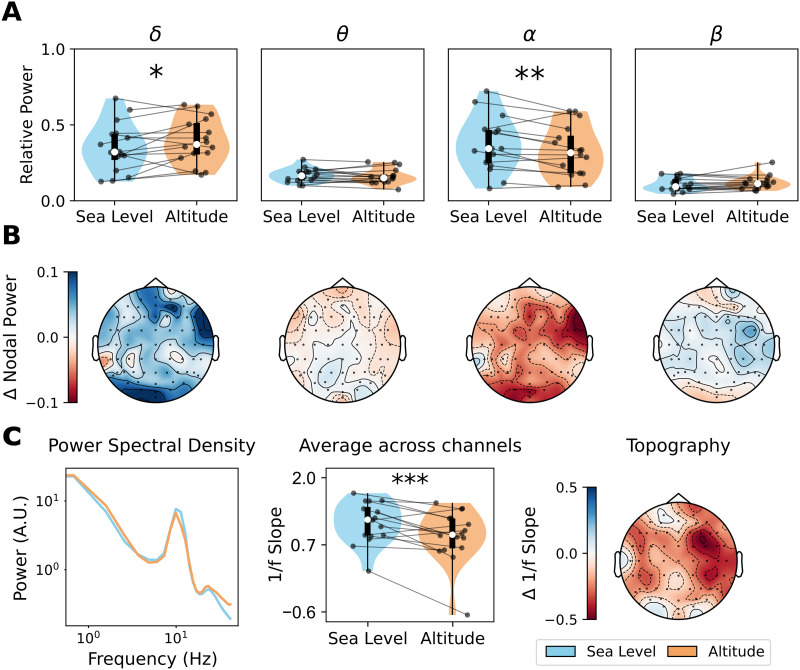
Changes in power spectrum by altitude. (A) Relative power, to broadband 0.5–30 Hz spectrum, in each frequency band. (B) Regional differences in nodal relative power (altitude minus sea level). (C) Power spectrum and 1/f slope averaged across channels; 1/f slope topography is also shown. Data points in violin plots correspond to subjects in both conditions: sea level and altitude. Box plots were built using the 1st and 3rd quartiles, the median, and the maximum and minimum values of distributions. All *p* values were FDR-corrected. ***p < 0.001, **p < 0.01, *p < 0.05, ∼*p* < 0.1.

To test if high-altitude hypoxia affected brain function, we first analyzed the effect of high-altitude conditions on the power spectrum of the EEG. We calculated the relative power in each frequency band. Results are presented in Figure 1, where we observed an increment of the *δ* band relative power in high altitude (*p* = 0.049, D = 0.277), and a reduction of the *α* band relative power (*p* = 0.003, D = −0.285), considering the average power across electrodes (Figure 1A). At a more local scale, we found an increase, by high altitude, of the *δ* band relative power in occipitoparietal scalp regions (*p* = 0.011, D = 0.385), and a decrease of *α* band relative power in occipitoparietal (*p* = 0.001, D = −0.336) and frontocentral (*p* = 0.006, D = −0.357) sensors (Figure 1B). No differences were found for *α* and *β* bands. Thus, results suggest that altitude-induced hypoxia concentrates the relative power in the slower bands of the EEG spectrum. Furthermore, changes in the *α* and *δ* bands are localized at both the posterior and anterior and only posterior scalp sensors, respectively.

Next, we calculated the aperiodic component of the PSD. Spectral parametrization showed a global decrease of 1/f slope driven by high-altitude hypoxia (Figure 1C, *p* = 0.0001, D = −0.42). When grouping channels across topographic regions, we found that the effect of high altitude on 1/f slope was significant for frontal (*p* = 0.012, D = −0.483), frontocentral (*p* = 0.0001, D = −0.700), centroparietal (*p* = 0.0001, D = −0.665), and temporal scalp regions (*p* = 0.0001, D = −0.587), but not for occipitoparietal sensors (*p* = 0.222, D = −0.222), similar to the topographic distribution of the effect of aging in aperiodic activity recently reported (Merkin et al., 2023).

### Global Functional Connectivity Was Altered by High-Altitude Hypoxia

**Figure 2.**
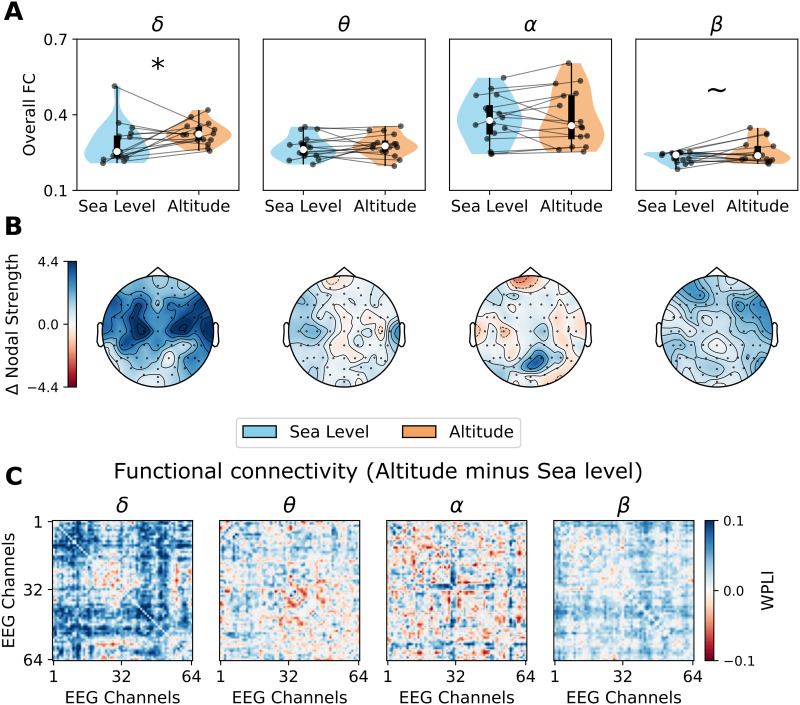
Functional connectivity (FC) strength in high-altitude hypoxia. (A) Overall FC, computed as the average of wPLI FC matrices per subject and for each frequency band. (B) Regional differences in nodal strength (altitude minus sea level). (C) Pairwise difference (altitude minus sea level) between FCs at each frequency band. Data points in violin plots correspond to subjects in both conditions: sea level and altitude. Box plots were built using the 1st and 3rd quartiles, the median, and the maximum and minimum values of distributions. All *p* values were FDR-corrected. ***p < 0.001, **p < 0.01, *p < 0.05, ∼*p* < 0.1.

To explore if high-altitude hypoxia produced altered brain patterns on functional networks, we computed the FC matrices in each frequency band using wPLI, firstly characterizing FC overall strength and then network topology. The results, presented in Figure 2, showed an increase in the global connectivity, named overall FC (Figure 2A) in the *δ* band (*p* = 0.048, D = 0.653), and a trend to be higher in the *β* band (*p* = 0.075, D = 0.644). At the local level, we computed the difference—altitude minus sea level—in nodal strength (defined as the weighted degree), using the weighted and nonthresholded FC matrices. We found that *δ* nodal strength was greater at altitude in temporal (*p* = 0.039, D = 0.805) and frontocentral scalp regions (*p* = 0.039, D = 0.946). No differences were found for the remaining frequency bands.

Qualitatively, the global increase in overall FC can be observed by visual inspection of the FC matrices at sea level and 4,000 masl (high altitude) (Figure 2C), where the contrast between the *δ* band FCs can be observed. This overall increase of *δ* band FC strength has been observed in several conditions, such as deep sleep (Imperatori et al., 2019) and anesthesia (Lee et al., 2017), where arousal levels abruptly decreased.

### Changes in Functional Network Topology Triggered by Altitude

We studied how high-altitude hypoxia affects the functional network topology of the thresholded and binarized FC matrices. Specifically, we computed the global efficiency (integration) and transitivity (segregation) of the thresholded networks. We computed the AUC values for the range of thresholds employed (from 5% to 40%).

**Figure 3.**
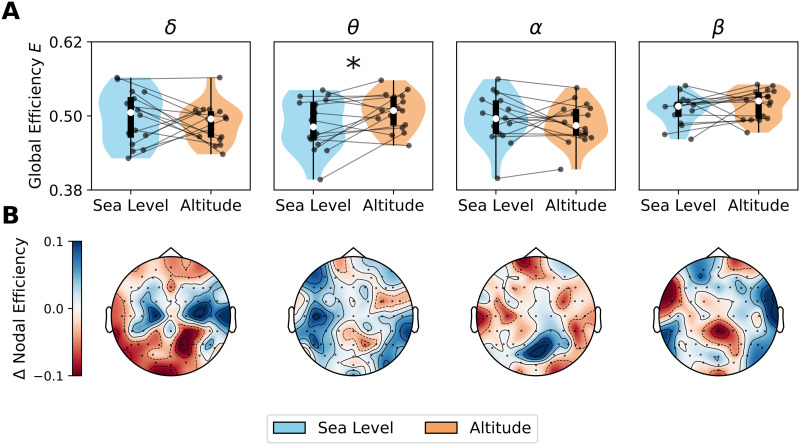
Global and local changes in integration by altitude. (A) Global efficiency, computed by numerical integration of network metric as function of the threshold. (B) Regional differences in nodal efficiency (altitude minus sea level). Data points in violin plots correspond to subjects in both conditions: sea level and altitude. Box plots were built using the 1st and 3rd quartiles, the median, and the maximum and minimum values of distributions. All *p* values were FDR-corrected. ***p < 0.001, **p < 0.01, *p < 0.05, ∼*p* < 0.1.

With respect to integration, we observed an increase of global efficiency in the *θ* band only at altitude versus sea level (*p* = 0.013, D = 0.594) (Figure 3A). Although we did not find differences in the *δ*, *α*, and *β* bands, it is possible that the changes in functional network topology are more localized rather than global. Consistent with this, we found regional changes in the *δ* band, where nodal efficiency decreased by altitude in the occipitoparietal sensors (*p* = 0.045, D = −0.870). In the *θ* band, nodal efficiency was higher in the centroparietal (*p* = 0.048, D = 0.618) regions. We did not find any local changes in the *α* and *β* bands. The local difference (altitude minus seal level) in nodal efficiency is presented in the head maps of Figure 3B.

**Figure 4.**
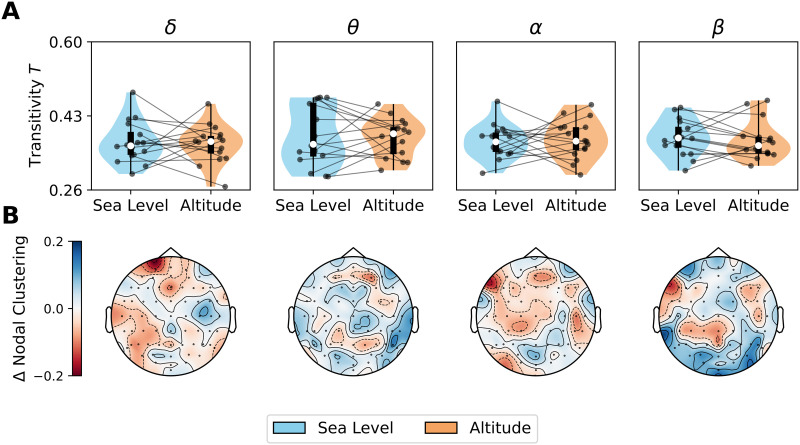
Global and local changes in segregation by altitude. (A) Transitivity, computed by numerical integration of network metric as function of the threshold. (B) Regional differences in nodal clustering coefficient (altitude minus sea level). Data points in violin plots correspond to subjects in both conditions: sea level and altitude. Box plots were built using the 1st and 3rd quartiles, the median, and the maximum and minimum values of distributions. All *p* values were FDR-corrected. ***p < 0.001, **p < 0.01, *p < 0.05, ∼*p* < 0.1.

Regarding segregation, we did not find any change in transitivity by altitude in all frequency bands (Figure 4A). However, we repeated the local network analysis to calculate the nodal clustering coefficient. In the *θ* band, high-altitude conditions produced an increase in the nodal clustering coefficient in the temporal scalp regions (*p* = 0.007, D = 0.529). In the *β* band, an increase in clustering triggered by altitude was found in occipital regions (*p* = 0.0001, D = 1.156). No local differences were observed in the *δ* and *α* bands. The local difference (altitude minus seal level) in nodal clustering is presented in the topoplots of Figure 4B.

### Hypoxia-Related Effects on Brain Function as a Model of Reversible Aging

**Figure 5.**
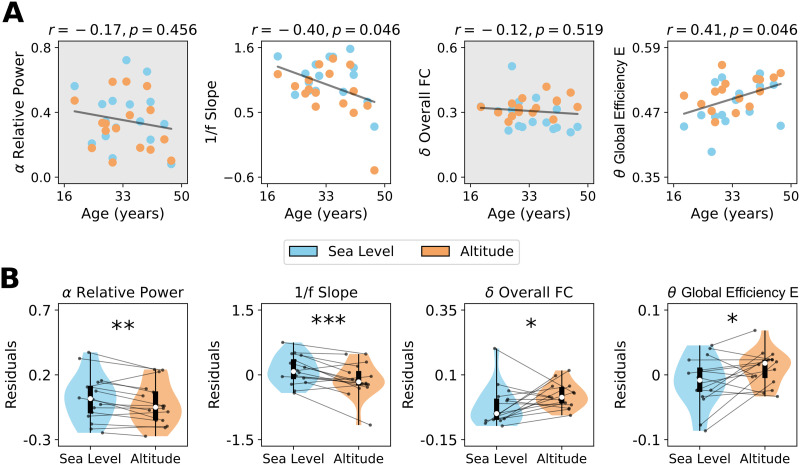
Effect of aging on brain activity. (A) Correlation of single subject age with *α* band relative power, 1/f slope, *δ* band overall FC (computed as the average of wPLI FC matrices), and *θ* band global efficiency (functional integration). Pearson’s *r* coefficients and *p* values are shown in the above figures. (B) Residuals obtained regressing out the effect of age from the set of EEG-related metrics. Data points in violin plots and scatter plots correspond to subjects in both conditions: sea level and altitude. Box plots were built using the 1st and 3rd quartiles, the median, and the maximum and minimum values of distributions. All *p* values were FDR-corrected. ***p < 0.001, **p < 0.01, *p < 0.05, ∼*p* < 0.1.

In the previous sections, we reported how high-altitude hypoxia impacts normal brain activity and connectivity. We proposed that these changes might be analogous with aging and neurodegeneration. To connect hypoxia-induced alterations more directly with brain aging, we correlated subjects’ chronological age with our EEG data measurements. To minimize the risk of multiple comparisons and consequently reduce potential false positives, we concentrated on the primary findings within each subsection, specifically: *α* band relative power, 1/f slope, *δ* band overall FC, and *θ* band integration (Figure 5A). Pooling data from both sea level and altitude conditions, we first identified a negative correlation between the 1/f slope and age (*r* = −0.40, *p* = 0.046), aligning with previously reported findings in the field (Merkin et al., 2023). Conversely, *θ* band global efficiency (integration) showed a positive correlation with age (*r* = 0.41, *p* = 0.046). These results imply that both the 1/f slope and *θ* band integration effectively capture changes characteristic of healthy aging, potentially indicating brain activity marked by increased electrophysiological noise and enhanced global functional integration.

It is possible that the changes ascribed to hypoxia can be influenced by participants’ age. To separate the effects of age from the EEG measurements, we regressed out the effect of age. (Figure 5B). We found that the results remained robust when controlling for age, observing a decrease in the *α* band relative power (*p* = 0.001, D = −0.289), a reduction of the 1/f slope (*p* = 0.0001, D = −0.638), an increase in *δ* band overall FC (*p* = 0.038, D = 0.658) and in *θ* band global efficiency (*p* = 0.017, D = 0.658) at high altitude compared to sea level. As such, the alterations triggered by hypoxia not only are observed when controlling for chronological age, but also followed the same direction as the effects observed in aging.

## DISCUSSION

The brain requires an ongoing and stable oxygen supply to support its underlying functions. The role of oxygen in the functional activity of the brain and its relationship with aging and its pathologies has been recently highlighted (Li et al., 2016). Metabolism is known to modulate brain function and neural activity (Medel et al., 2022), especially during aging (Deery et al., 2023), and metabolic biomarkers are widely used to diagnose age-related brain pathologies (Chételat et al., 2020). We extended this knowledge by isolating the effect of oxygen supply on brain function, thus controlling for other processes involved in brain development and neurodegeneration. Our results suggest that canonical age-related effects on functional EEG activity can be simulated by inducing hypoxia (in this case using high altitude; 4,000 masl).

We found that at high altitude the relative power of the slower oscillations were increased, while *α* band relative power decreased compared to sea level. Convergently, we observed a widespread reduction of 1/f slope (flattened spectrum) at altitude, which suggests that hypoxia increases neural (electrophysiological) noise (Voytek et al., 2015). Thus, our spectral analysis suggests a shift to slower and more noisy brain activity at high altitude.

With respect to functional connectivity, we found an increase in overall FC at high altitude (global frequency synchronization) in *δ* band. Regarding topology, global integration was higher in high altitude in the *θ* band compared to sea level. The results suggest a shift to a more integrated functional network topology at high altitude. At the local level, changes in nodal properties were region-specific: occipital sensors showed a decrease in their integration, with the opposite observed for central sensors. Finally, we found a correlation between chronological age, 1/f slope and *θ* band functional integration, demonstrating the potential parallels between hypoxia and brain aging. Together these results indicate that high-altitude hypoxia affects specific EEG bands and network metrics, suggesting potential biomarkers to identify and study the effects of high-altitude hypoxia on the brain. Furthermore, changes seen in brain activity due to hypoxia were reminiscent of those observed across age, which suggests that hypoxia may serve as a model for aging or neurodegeneration.

Our results are consistent with previous reports (Babiloni et al., 2014) in which an increase in the *δ* and *θ* relative power was triggered by hypoxia, disrupting the normal *α* rhythms observed in RS-EEG, especially in the occipitoparietal scalp regions (Lozano-Soldevilla, 2018; Mandal et al., 2018). High-altitude hypoxia might produce deficits in vasomotor reactivity and neurovascular coupling, decoupling thalamocortical networks, and ultimately generating the slow rhythms observed by high altitude (Babiloni et al., 2014). Similar effects are observed as a result of GABAergic anesthetics (Ching et al., 2010; Purdon et al., 2013) and dissociative drugs (Akeju et al., 2016; Vlisides et al., 2017); whereas pro-cholinergic drugs, usually associated with increased attention and arousal (Coronel-Oliveros et al., 2023; Giessing et al., 2013; Hasselmo & Sarter, 2011; Honey et al., 2017), produce an opposing profile of effects (see Lozano-Soldevilla, 2018). Furthermore, the spectral analysis revealed an increase of the 1/f slope at altitude compared to sea level. In line with previous works in aging and Alzheimer’s disease (Martínez-Cañada et al., 2023; Voytek et al., 2015; Vyšata et al., 2014), this suggests a shift toward higher electrophysiological noise by high-altitude hypoxia. This increase in neural noise is linked to desynchronized spiking activity, disrupting network communication, and ultimately producing cognitive deficits (Voytek & Knight, 2015). Furthermore, the higher 1/f slope generated by high altitude was stronger in fronto-parietal scalp regions. These results are consistent both with the cognitive deficits triggered by high altitude (Asmaro et al., 2013; de Aquino Lemos et al., 2012; Malle et al., 2016) and the role of the fronto-parietal network in supporting attention and executive functions (Marek & Dosenbach, 2018).

Network analysis at the global level revealed FC alterations triggered by hypoxia. Firstly, using wPLI, similar results were reported for different states of arousal and consciousness increases in *δ* median wPLI (overall FC), and a decrease in *α* median wPLI in N3 sleep (Imperatori et al., 2019) and propofol anesthesia (Lee et al., 2017). High-altitude hypoxia also produced a global reconfiguration of brain networks, reflected in the shift to functional integration produced within the *θ* band. Although the brain “integrates” functions during some types of behavioral tasks, usually in tasks with a high cognitive load (Shine & Poldrack, 2018), this may be undesirable in resting-state conditions (Wig, 2017). Some examples where a reduction of RS segregation (or an increase in integration) have been linked to deficits or impairments in brain functions include childhood-onset schizophrenia (Alexander-Bloch et al., 2010), cognitive decline in healthy aging (Pedersen et al., 2021; Song et al., 2014), Alzheimer’s disease, mild cognitive impairment (Brier et al., 2014; Franciotti et al., 2019; Sanz-Arigita et al., 2010; Seo et al., 2013), and obstructive sleep apnea (Luo et al., 2015). Alzheimer’s disease and obstructive sleep apnea are of particular interest, as the slowing of RS-EEG rhythms observed during hypoxia were also observed in these cases (Babiloni et al., 2014). Furthermore, they are associated with impairments in vasomotor reactivity and neurovascular coupling (Li et al., 2020; Smoliński & Członkowska, 2016), as observed in acute mountain sickness produced by high-altitude conditions (Jansen et al., 1999).

At the local level, differences in functional network changes were observed between high altitude and sea level. The local increase of segregation (or the reduced integration) in temporal and occipital scalp regions may reflect a form of “sensory disconnection” within the brain, considering the role of highly integrated nodes, as the “rich club” brain regions, in supporting long-range network connectivity (Faber et al., 2019). On the other hand, both the increment of *δ* overall FC in frontal and central scalp regions, and the increase of integration (*θ* band) in centro-parietal regions suggest a strengthening of frontocentral connectivity. It is hypothesized that the faster oscillations are involved in local connectivity, and the slower ones in long-range connectivity (Harmony, 2013; Niknazar et al., 2022). Interestingly, some of the changes in functional integration (increased global efficiency and reduced occipitoparietal nodal efficiecy) were observed in the slowest frequency bands (*δ* and *θ*), while the increase in occipitoparietal segregation occurred in the faster *β* band, suggesting that the band-specific changes caused by altitude in network topology and FC respond to different spatial scales of signal processing.

The comparable EEG profiles observed in relation to age and high-altitude conditions underscore the importance of oxygen metabolism in understanding the trajectory of brain health across life-span. Moreover, if the patterns in EEG profiles observed across ages are found to be robust, they may offer potential avenues for early detection of age-related brain alterations. Identifying these shifts may facilitate timely interventions aimed at maintaining or even enhancing cognitive health. Further investigations may focus on understanding the mechanisms behind these parallels between high-altitude hypoxia and aging. Potential questions of interest include whether other factors, such as genetic predispositions or environmental variables modulate the observed effects.

Our study has some limitations that constrain the impact of these findings: Firstly, the influence of high altitude on the EEG functional connectivity could comprise not only the reduction of the oxygen pressure by altitude, but also changes in environmental variables. For example, external temperature may affect EEG spectral properties (de Labra et al., 2021; Gaenshirt et al., 1954; Pearcy & Virtue, 1959). Our results must be contrasted by conditions in which environmental variables can be controlled, as usually done when simulating hypobaric hypoxia.

Secondly, it is worth noting that the findings presented here are based on a limited sample size. Future research with larger cohorts is crucial to validate and extend these preliminary observations. Despite this limitation, the study lays the groundwork for more extensive investigations into the intricate relationships between brain activity, aging, and environmental stressors such as high-altitude hypoxia.

Since the computation of EEG spectral features and FC was limited to the sensor space, our analyses precluded direct associations between EEG features and brain regions. However, it has been suggested that networks built from the sensors space can capture the rich structure of brain interactions, considering the effects of volume conduction and even when controlled for them (Schaworonkow & Nikulin, 2022). Furthermore, the decision of estimating EEG connectivity at either the sensors or source level remains arbitrary unless specific hypotheses on regions-of-interest are proposed (Lai et al., 2018). Inverse methods for source reconstruction can introduce variability in network and connectivity estimates (Mahjoory et al., 2017), and sensor-level topological properties have been reported as highly consistent, robust, and stable in the long term (Chu et al., 2012). Finally, it is essential to note that EEG connectivity highly relies on the connectivity metric selected for the analysis (Perera et al., 2020; Prado et al., 2022; Wang et al., 2014). In this study, the reason for using wPLI was to discharge spurious short-range functional connections that can be obtained in high-density electrode layouts due to head volume conduction. Future studies can implement EEG connectivity metrics that can detect physiological close-to-zero lag interactions while reducing the impact of head volume conduction (Sanchez Bornot et al., 2018). Alternatively, future work can employ composite metrics of connectivity, that is, machine-learning approaches that integrate information provided by different connectivity metrics, which has been successful in estimating brain aging (Mohanty et al., 2020) and classifying neurodegenerative diseases (Prado et al., 2023a, 2023b).

In summary, this work suggests that alterations in brain metabolism via acutely manipulating oxygen supply through high-altitude hypoxia systematically affect brain function and its organization. The alterations triggered by high altitude may be utilized as biomarkers related to hypoxia. Therefore, such findings may help to advise and evidence treatments and therapies that aim to recover normal brain function. Furthermore, our results suggest that altered oxygen metabolism may be an underlying mechanism of the functional changes observed in developmental and pathological aging. By comparing conditions in which oxygen availability differs, we show that hypoxia alone is sufficient to alter EEG spectral and network properties of the brain, replicating previous results seen in aging and dementia. Further research should explore whether the reported relation has an impact on cognitive decline and behavior. The functional and cognitive relevance of oxygen supply underscores the need for studies analyzing the role of metabolic decline in brain physiological and pathological processes.

## ACKNOWLEDGMENTS

We want to thank all subjects who kindly participated in the experiments.

## AUTHOR CONTRIBUTIONS

Carlos Coronel-Oliveros: Formal analysis; Investigation; Methodology; Validation; Visualization; Writing – original draft; Writing – review & editing. Vicente Medel: Formal analysis; Investigation; Methodology; Validation; Writing – original draft; Writing – review & editing. Grace Whitaker: Formal analysis; Methodology; Validation; Visualization; Writing – original draft; Writing – review & editing. Aland Astudillo: Data curation; Investigation; Methodology; Validation; Writing – original draft; Writing – review & editing. David Gallagher: Conceptualization; Investigation; Writing – original draft; Writing – review & editing. Lucía Z-Rivera: Data curation; Methodology; Writing – original draft; Writing – review & editing. Pavel Prado: Conceptualization; Writing – review & editing. Wael El-Deredy: Conceptualization; Funding acquisition; Investigation; Project administration; Resources; Supervision; Writing – review & editing. Patricio Orio: Investigation; Methodology; Resources; Supervision; Writing – review & editing. Alejandro Weinstein: Conceptualization; Funding acquisition; Investigation; Project administration; Resources; Supervision; Writing – review & editing.

## FUNDING INFORMATION

Vicente Medel, Agencia Nacional de Investigación y Desarrollo (https://dx.doi.org/10.13039/501100020884), Award ID: ANID/Fondecyt 3230557. Alejandro Weinstein, Agencia Nacional de Investigación y Desarrollo (https://dx.doi.org/10.13039/501100020884), Award ID: ANID/Fondef Id16i10322 and ANID/Fondecyt 1231132. Patricio Orio, Agencia Nacional de Investigación y Desarrollo (https://dx.doi.org/10.13039/501100020884), Award ID: ANID/Fondecyt 1211750.

## DATA AND CODE AVAILABILITY

This work did not produce original codes for data analysis and simulations. Functional connectivity was assessed through MNE for Python (https://github.com/mne-tools; Gramfort et al., 2013). Functional network analysis was performed using the brain connectivity toolbox (https://github.com/fiuneuro/brainconn; Rubinov & Sporns, 2010). For the 1/f analysis, we used the FOOOF library in Python (https://github.com/fooof-tools/fooof; Donoghue et al., 2020). Data is available on request from Dr. Alejandro Weinstein, University of Valparaíso (email: alejandro.weinstein@uv.cl). A formal data sharing agreement is required.

## TECHNICAL TERMS

One over F (1/f): Describes the distribution of power (or energy) across different frequencies in the EEG signal. The power at a given frequency is inversely proportional to that frequency.
Functional connectivity: Statistical relationships between pairs of neurons, brain regions or networks. Usually measured using the time series of brain activity (spikes, EEG, fMRI BOLD) and a measure of brain connectivity (e.g., Pearson’s correlation).
Network: A mathematical representation of a system of elements. In the case of the brain, neurons and brain areas consist of nodes connected by edges, represented by anatomical or functional links.
Functional connectivity matrix: A matrix that captures the functional connectivity values between all the possible pairs of regions in a network.
Integration: Property of a network that allows the communication and information transfer between specialized, distant, and locally connected nodes.
Segregation: The degree of local connectivity within a network.
Overall FC: Mean values (the average) of the functional connectivity matrices, generally named global correlations.

## References

[bib1] Akeju, O., Song, A. H., Hamilos, A. E., Pavone, K. J., Flores, F. J., Brown, E. N., & Purdon, P. L. (2016). Electroencephalogram signatures of ketamine anesthesia-induced unconsciousness. Clinical Neurophysiology, 127(6), 2414–2422. 10.1016/j.clinph.2016.03.005, 27178861 PMC4871620

[bib2] Alexander-Bloch, A. F., Gogtay, N., Meunier, D., Birn, R., Clasen, L., Lalonde, F., … Bullmore, E. T. (2010). Disrupted modularity and local connectivity of brain functional networks in childhood-onset schizophrenia. Frontiers in Systems Neuroscience, 4, 147. 10.3389/fnsys.2010.00147, 21031030 PMC2965020

[bib3] Asmaro, D., Mayall, J., … Ferguson, S. (2013). Cognition at altitude: Impairment in executive and memory processes under hypoxic conditions. Aviation, Space, and Environmental Medicine, 84(11), 1159–1165. 10.3357/ASEM.3661.2013, 24279229

[bib4] Babiloni, C., Del Percio, C., Lizio, R., Infarinato, F., Blin, O., Bartres-Faz, D., … Richardson, J. C. (2014). A review of the effects of hypoxia, sleep deprivation and transcranial magnetic stimulation on EEG activity in humans: Challenges for drug discovery for Alzheimer’s disease. Current Alzheimer Research, 11(5), 501–518. 10.2174/1567205011666140317095623, 24635844

[bib5] Benjamini, Y., & Hochberg, Y. (1995). Controlling the false discovery rate: A practical and powerful approach to multiple testing. Journal of The Royal Statistical Society: Series B (Methodological), 57(1), 289–300. 10.1111/j.2517-6161.1995.tb02031.x

[bib6] Brier, M. R., Thomas, J. B., Fagan, A. M., Hassenstab, J., Holtzman, D. M., Benzinger, T. L., … Ances, B. M. (2014). Functional connectivity and graph theory in preclinical Alzheimer’s disease. Neurobiology of Aging, 35(4), 757–768. 10.1016/j.neurobiolaging.2013.10.081, 24216223 PMC3880636

[bib8] Chételat, G., Arbizu, J., Barthel, H., Garibotto, V., Law, I., Morbelli, S., … Drzezga, A. (2020). Amyloid-PET and ^18^F-FDG-PET in the diagnostic investigation of Alzheimer’s disease and other dementias. The Lancet Neurology, 19(11), 951–962. 10.1016/S1474-4422(20)30314-8, 33098804

[bib9] Ching, S., Cimenser, A., Purdon, P. L., Brown, E. N., & Kopell, N. J. (2010). Thalamocortical model for a propofol-induced *α*-rhythm associated with loss of consciousness. Proceedings of the National Academy of Sciences, 107(52), 22665–22670. 10.1073/pnas.1017069108, 21149695 PMC3012501

[bib10] Chiu, H.-Y., Wang, M.-Y., Chang, C.-K., Chen, C.-M., Chou, K.-R., Tsai, J.-C., & Tsai, P.-S. (2014). Early morning awakening and nonrestorative sleep are associated with increased minor non-fatal accidents during work and leisure time. Accident Analysis & Prevention, 71, 10–14. 10.1016/j.aap.2014.05.002, 24875435

[bib11] Chu, C. J., Kramer, M. A., Pathmanathan, J., Bianchi, M. T., Westover, M. B., Wizon, L., & Cash, S. S. (2012). Emergence of stable functional networks in long-term human electroencephalography. Journal of Neuroscience, 32(8), 2703–2713. 10.1523/JNEUROSCI.5669-11.2012, 22357854 PMC3361717

[bib12] Coronel-Oliveros, C., Giessing, C., Medel, V., Cofré, R., & Orio, P. (2023). Whole-brain modeling explains the context-dependent effects of cholinergic neuromodulation. NeuroImage, 265, 119782. 10.1016/j.neuroimage.2022.119782, 36464098

[bib13] de Aquino Lemos, V., Antunes, H. K. M, dos Santos, R. V. T., Lira, F. S., Tufik, S., & de Mello, M. T. (2012), High altitude exposure impairs sleep patterns, mood, and cognitive functions. Psychophysiology, 49(9), 1298–1306. 10.1111/j.1469-8986.2012.01411.x, 22803634

[bib14] Delorme, A., & Makeig, S. (2004). EEGLAB: An open source toolbox for analysis of single-trial EEG dynamics including independent component analysis. Journal of Neuroscience Methods, 134(1), 9–21. 10.1016/j.jneumeth.2003.10.009, 15102499

[bib15] de Labra, C., Pardo-Vazquez, J. L., Cudeiro, J., & Rivadulla, C. (2021). Hyperthermia-induced changes in EEG of anesthetized mice subjected to passive heat exposure. Frontiers in Systems Neuroscience, 15, 709337. 10.3389/fnsys.2021.709337, 34566589 PMC8458808

[bib16] Deery, H. A., Di Paolo, R., Moran, C., Egan, G. F., & Jamadar, S. D. (2023). Lower brain glucose metabolism in normal ageing is predominantly frontal and temporal: A systematic review and pooled effect size and activation likelihood estimates meta-analyses. Human Brain Mapping, 44(3), 1251–1277. 10.1002/hbm.26119, 36269148 PMC9875940

[bib17] Donoghue, T., Haller, M., Peterson, E. J., Varma, P., Sebastian, P., Gao, R., … Voytek, B. (2020). Parameterizing neural power spectra into periodic and aperiodic components. Nature Neuroscience, 23(12), 1655–1665. 10.1038/s41593-020-00744-x, 33230329 PMC8106550

[bib18] Faber, S. P., Timme, N. M., Beggs, J. M., & Newman, E. L. (2019). Computation is concentrated in rich clubs of local cortical networks. Network Neuroscience, 3(2), 384–404. 10.1162/netn_a_00069, 30793088 PMC6370472

[bib19] Franciotti, R., Falasca, N. W., Arnaldi, D., Famà, F., Babiloni, C., Onofrj, M., … Bonanni, L. (2019). Cortical network topology in prodromal and mild dementia due to Alzheimer’s disease: Graph theory applied to resting state EEG. Brain Topography, 32(1), 127–141. 10.1007/s10548-018-0674-3, 30145728 PMC6326972

[bib20] Fraschini, M., Demuru, M., Crobe, A., Marrosu, F., Stam, C. J., & Hillebrand, A. (2016). The effect of epoch length on estimated EEG functional connectivity and brain network organisation. Journal of Neural Engineering, 13(3), 036015. 10.1088/1741-2560/13/3/036015, 27137952

[bib21] Gaenshirt, H., Krenkel, W., & Zylka, W. (1954). The electrocorticogram of the cat’s brain at temperatures between 40°C and 20°C. Electroencephalography and Clinical Neurophysiology, 6, 409–413. 10.1016/0013-4694(54)90055-3, 13200412

[bib22] Gao, R., Peterson, E. J., & Voytek, B. (2017). Inferring synaptic excitation/inhibition balance from field potentials. NeuroImage, 158, 70–78. 10.1016/j.neuroimage.2017.06.078, 28676297

[bib23] Gatica, M., Cofré, R., Mediano, P. A. M., Rosas, F. E., Orio, P., Diez, I., … Cortes, J. M. (2021). High-order interdependencies in the aging brain. Brain Connectivity, 11(9), 734–744. 10.1089/brain.2020.0982, 33858199

[bib24] Gatica, M., Rosas, F. E., Mediano, P. A. M., Diez, I., Swinnen, S. P., Orio, P., … Cortes, J. M. (2022). High-order functional redundancy in ageing explained via alterations in the connectome in a whole-brain model. PLoS Computational Biology, 18(9), e1010431. 10.1371/journal.pcbi.1010431, 36054198 PMC9477425

[bib25] Giessing, C., Thiel, C. M., Alexander-Bloch, A. F., Patel, A. X., & Bullmore, E. T. (2013). Human brain functional network changes associated with enhanced and impaired attentional task performance. Journal of Neuroscience, 33(14), 5903–5914. 10.1523/JNEUROSCI.4854-12.2013, 23554472 PMC6618923

[bib26] Ginestet, C. E., Nichols, T. E., Bullmore, E. T., & Simmons, A. (2011). Brain network analysis: Separating cost from topology using cost-integration. PLoS One, 6(7), e21570. 10.1371/journal.pone.0021570, 21829437 PMC3145634

[bib27] Gramfort, A., Luessi, M., Larson, E., Engemann, D. A., Strohmeier, D., Brodbeck, C., … Hämäläinen, M. (2013). MEG and EEG data analysis with MNE-Python. Frontiers in Neuroscience, 7, 267. 10.3389/fnins.2013.00267, 24431986 PMC3872725

[bib28] Harmony, T. (2013). The functional significance of delta oscillations in cognitive processing. Frontiers in Integrative Neuroscience, 7, 83. 10.3389/fnint.2013.00083, 24367301 PMC3851789

[bib29] Hasselmo, M. E., & Sarter, M. (2011). Modes and models of forebrain cholinergic neuromodulation of cognition. Neuropsychopharmacology, 36(1), 52–73. 10.1038/npp.2010.104, 20668433 PMC2992803

[bib30] Honey, C. J., Newman, E. L., & Schapiro, A. C. (2017). Switching between internal and external modes: A multiscale learning principle. Network Neuroscience, 1(4), 339–356. 10.1162/NETN_a_00024, 30090870 PMC6063714

[bib31] Hu, S. L., Shi, J. T., Xiong, W., Li, W. N., Fang, L. C., & Feng, H. (2017). Oxiracetam or fastigial nucleus stimulation reduces cognitive injury at high altitude. Brain and Behavior, 7(10), e00762. 10.1002/brb3.762, 29075554 PMC5651378

[bib32] Imperatori, L. S., Betta, M., Cecchetti, L., Canales-Johnson, A., Ricciardi, E., Siclari, F., … Bernardi, G. (2019). EEG functional connectivity metrics wPLI and wSMI account for distinct types of brain functional interactions. Scientific Reports, 9(1), 8894. 10.1038/s41598-019-45289-7, 31222021 PMC6586889

[bib33] Jansen, G. F., Krins, A., & Basnyat, B. (1999). Cerebral vasomotor reactivity at high altitude in humans. Journal of Applied Physiology, 86(2), 681–686. 10.1152/jappl.1999.86.2.681, 9931208

[bib34] Kida, T., Tanaka, E., & Kakigi, R. (2016). Multi-dimensional dynamics of human electromagnetic brain activity. Frontiers in Human Neuroscience, 9, 713. 10.3389/fnhum.2015.00713, 26834608 PMC4717327

[bib35] King, B. R., van Ruitenbeek, P., Leunissen, I., Cuypers, K., Heise, K.-F., Santos Monteiro, T., … Swinnen, S. P. (2018). Age-related declines in motor performance are associated with decreased segregation of large-scale resting state brain networks. Cerebral Cortex, 28(12), 4390–4402. 10.1093/cercor/bhx297, 29136114 PMC6215458

[bib36] Lai, M., Demuru, M., Hillebrand, A., & Fraschini, M. (2018). A comparison between scalp- and source-reconstructed EEG networks. Scientific Reports, 8(1), 12269. 10.1038/s41598-018-30869-w, 30115955 PMC6095906

[bib37] Latora, V., & Marchiori, M. (2001). Efficient behavior of small-world networks. Physical Review Letters, 87(19), 198701. 10.1103/PhysRevLett.87.198701, 11690461

[bib38] Lee, M., Sanders, R. D., Yeom, S.-K., Won, D.-O., Seo, K.-S., Kim, H. J., … Lee, S.-W. (2017). Network properties in transitions of consciousness during propofol-induced sedation. Scientific Reports, 7(1), 16791. 10.1038/s41598-017-15082-5, 29196672 PMC5711919

[bib39] Lendner, J. D., Helfrich, R. F., Mander, B. A., Romundstad, L., Lin, J. J., Walker, M. P., … Knight, R. T. (2020). An electrophysiological marker of arousal level in humans. eLife, 9, e55092. 10.7554/eLife.55092, 32720644 PMC7394547

[bib40] Li, G., Zhang, T., Chen, X., Shang, C., & Wang, Y. (2016). Effect of intermittent hypoxic training on hypoxia tolerance based on brain functional connectivity. Physiological Measurement, 37(12), 2299–2316. 10.1088/1361-6579/37/12/2299, 27897151

[bib41] Li, N., Liu, Y., Zhao, Y., Wu, X., Tong, J., & Hua, Y. (2020). Cerebrovascular reactivity in young and old patients with obstructive sleep apnea. Sleep Medicine, 73, 125–129. 10.1016/j.sleep.2020.04.029, 32827884

[bib42] Lozano-Soldevilla, D. (2018). On the physiological modulation and potential mechanisms underlying parieto-occipital alpha oscillations. Frontiers in Computational Neuroscience, 12, 23. 10.3389/fncom.2018.00023, 29670518 PMC5893851

[bib43] Luo, Y. G., Wang, D., Liu, K., Weng, J., Guan, Y., Chan, K. C., … Shi, L. (2015). Brain structure network analysis in patients with obstructive sleep apnea. PLoS One, 10(9), e0139055. 10.1371/journal.pone.0139055, 26413809 PMC4587669

[bib44] Mahjoory, K., Nikulin, V. V., Botrel, L., Linkenkaer-Hansen, K., Fato, M. M., & Haufe, S. (2017). Consistency of EEG source localization and connectivity estimates. NeuroImage, 152, 590–601. 10.1016/j.neuroimage.2017.02.076, 28300640

[bib45] Malle, C., Ginon, B., & Bourrilhon, C. (2016). Brief working memory and physiological monitoring during a high-altitude expedition. High Altitude Medicine & Biology, 17(4), 359–364. 10.1089/ham.2016.0022, 27548274

[bib46] Mandal, P. K., Banerjee, A., Tripathi, M., & Sharma, A. (2018). A comprehensive review of magnetoencephalography (MEG) studies for brain functionality in healthy aging and Alzheimer’s disease (AD). Frontiers in Computational Neuroscience, 12, 60. 10.3389/fncom.2018.00060, 30190674 PMC6115612

[bib47] Marek, S., & Dosenbach, N. U. F. (2018). The frontoparietal network: Function, electrophysiology, and importance of individual precision mapping. Dialogues in Clinical Neuroscience, 20(2), 133–140. 10.31887/DCNS.2018.20.2/smarek, 30250390 PMC6136121

[bib48] Martínez-Cañada, P., Perez-Valero, E., Minguillon, J., Pelayo, F., López-Gordo, M. A., & Morillas, C. (2023). Combining aperiodic 1/f slopes and brain simulation: An EEG/MEG proxy marker of excitation/inhibition imbalance in Alzheimer’s disease. Alzheimer’s & Dementia, 15(3), e12477. 10.1002/dad2.12477, 37662693 PMC10474329

[bib49] Medel, V., Crossley, N., Gajardo, I., Muller, E., Barros, L. F., Shine, J. M., & Sierralta, J. (2022). Whole-brain neuronal MCT2 lactate transporter expression links metabolism to human brain structure and function. Proceedings of the National Academy of Sciences, 119(33), e2204619119. 10.1073/pnas.2204619119, 35939682 PMC9388117

[bib50] Medel, V., Irani, M., Crossley, N., Ossandón, T., & Boncompte, G. (2023). Complexity and 1/f slope jointly reflect brain states. Scientific Reports, 13(1), 21700. 10.1038/s41598-023-47316-0, 38065976 PMC10709649

[bib51] Merkin, A., Sghirripa, S., Graetz, L., Smith, A. E., Hordacre, B., Harris, R., … Goldsworthy, M. (2023). Do age-related differences in aperiodic neural activity explain differences in resting EEG alpha? Neurobiology of Aging, 121, 78–87. 10.1016/j.neurobiolaging.2022.09.003, 36379095

[bib52] Mohanty, R., Sethares, W. A., Nair, V. A., & Prabhakaran, V. (2020). Rethinking measures of functional connectivity via feature extraction. Scientific Reports, 10(1), 1298. 10.1038/s41598-020-57915-w, 31992762 PMC6987226

[bib53] Mostame, P., & Sadaghiani, S. (2020). Phase-and amplitude-coupling are tied by an intrinsic spatial organization but show divergent stimulus-related changes. NeuroImage, 219, 117051. 10.1016/j.neuroimage.2020.117051, 32540356

[bib54] Muza, S. R., Beidleman, B. A., & Fulco, C. S. (2010). Altitude preexposure recommendations for inducing acclimatization. High Altitude Medicine & Biology, 11(2), 87–92. 10.1089/ham.2010.1006, 20586592

[bib55] Newman, M. E. (2003). The structure and function of complex networks. SIAM Review, 45(2), 167–256. 10.1137/S003614450342480

[bib56] Nichols, T. E., & Holmes, A. P. (2002). Nonparametric permutation tests for functional neuroimaging: A primer with examples. Human Brain Mapping, 15(1), 1–25. 10.1002/hbm.1058, 11747097 PMC6871862

[bib57] Niknazar, H., Malerba, P., & Mednick, S. C. (2022). Slow oscillations promote long-range effective communication: The key for memory consolidation in a broken-down network. Proceedings of the National Academy of Sciences, 119(26), e2122515119. 10.1073/pnas.2122515119, 35733258 PMC9245646

[bib58] Ochi, G., Kanazawa, Y., Hyodo, K., Suwabe, K., Shimizu, T., Fukuie, T., Byun, K., & Soya, H. (2018). Hypoxia-induced lowered executive function depends on arterial oxygen desaturation. Journal of Physiological Sciences, 68(6), 847–853. 10.1007/s12576-018-0603-y, 29536370 PMC10717617

[bib59] Ozaki, H., Watanabe, S., & Suzuki, H. (1995). Topographic EEG changes due to hypobaric hypoxia at simulated high altitude. Electroencephalography and Clinical Neurophysiology, 94(5), 349–356. 10.1016/0013-4694(94)00311-8, 7774521

[bib60] Pearcy, W. C., & Virtue, R. W. (1959). The electroencephalogram in hypothermia with circulatory arrest. Anesthesiology, 20(3), 341–347. 10.1097/00000542-195905000-00014, 13650220

[bib61] Pedersen, R., Geerligs, L., Andersson, M., Gorbach, T., Avelar-Pereira, B., Wåhlin, A., … Salami, A. (2021). When functional blurring becomes deleterious: Reduced system segregation is associated with less white matter integrity and cognitive decline in aging. NeuroImage, 242, 118449. 10.1016/j.neuroimage.2021.118449, 34358662

[bib193] Perera, D., Wang, Y. K., Lin, C. T., Zheng, J., Nguyen, H. T., & Chai, R. (2020). Statistical analysis of brain connectivity estimators during distracted driving. In 2020 42nd Annual International Conference of the IEEE Engineering in Medicine & Biology Society (EMBC) (pp. 3208–3211). Montreal, QC, Canada. 10.1109/EMBC44109.2020.9176240 33018687

[bib62] Petrassi, F. A., Hodkinson, P. D., Walters, P. L., & Gaydos, S. J. (2012). Hypoxic hypoxia at moderate altitudes: Review of the state of the science. Aviation, Space, and Environmental Medicine, 83(10), 975–984. 10.3357/ASEM.3315.2012, 23066620

[bib63] Prado, P., Birba, A., Cruzat, J., Santamaría-García, H., Parra, M., Moguilner, S., Tagliazucchi, E., & Ibáñez, A. (2022). Dementia ConnEEGtome: Towards multicentric harmonization of EEG connectivity in neurodegeneration. International Journal of Psychophysiology, 172, 24–38. 10.1016/j.ijpsycho.2021.12.008, 34968581 PMC9887537

[bib64] Prado, P., Moguilner, S., Mejía, J. A., Sainz-Ballesteros, A., Otero, M., Birba, A., Santamaría-García, H., Legaz, A., Fittipaldi, S., Cruzat, J., Tagliazucchi, E., Parra, M., Herzog, R., & Ibáñez, A. (2023a). Source space connectomics of neurodegeneration: One-metric approach does not fit all. Neurobiology of Disease, 179, 106047. 10.1016/j.nbd.2023.106047, 36841423 PMC11170467

[bib65] Prado, P., Mejía, J. A., Sainz-Ballesteros, A., Birba, A., Moguilner, S., Herzog, R., Otero, M., Cuadros, J., Z-Rivera, L., O’Byrne, D. F., Parra, M., & Ibáñez, A. (2023b). Harmonized multi-metric and multi-centric assessment of EEG source space connectivity for dementia characterization. Alzheimer’s & Dementia, 15(3), e12455. 10.1002/dad2.12455, 37424962 PMC10329259

[bib66] Purdon, P. L., Pierce, E. T., Mukamel, E. A., Prerau, M. J., Walsh, J. L., Wong, K. F. K., … Brown, E. N. (2013). Electroencephalogram signatures of loss and recovery of consciousness from propofol. Proceedings of the National Academy of Sciences, 110(12), E1142–E1151. 10.1073/pnas.1221180110, 23487781 PMC3607036

[bib69] Rubinov, M., & Sporns, O. (2010). Complex network measures of brain connectivity: Uses and interpretations. NeuroImage, 52(3), 1059–1069. 10.1016/j.neuroimage.2009.10.003, 19819337

[bib70] Sanchez Bornot, J. M., Wong-Lin, K., Ahmad, A. L., & Prasad, G. (2018). Robust EEG/MEG based functional connectivity with the envelope of the imaginary coherence: Sensor space analysis. Brain Topography, 31(6), 895–916. 10.1007/s10548-018-0640-0, 29546509 PMC6182573

[bib71] Sanz-Arigita, E. J., Schoonheim, M. M., Damoiseaux, J. S., Rombouts, S. A., Maris, E., Barkhof, F., … Stam, C. J. (2010). Loss of ‘small-world’ networks in Alzheimer’s disease: Graph analysis of FMRI resting-state functional connectivity. PLoS One, 5(11), e13788. 10.1371/journal.pone.0013788, 21072180 PMC2967467

[bib72] Schaworonkow, N., & Nikulin, V. V. (2022). Is sensor space analysis good enough? Spatial patterns as a tool for assessing spatial mixing of EEG/MEG rhythms. NeuroImage, 253, 119093. 10.1016/j.neuroimage.2022.119093, 35288283

[bib73] Seo, E. H., Lee, D. Y., Lee, J. M., Park, J. S., Sohn, B. K., Lee, D. S., … Woo, J. I. (2013). Whole-brain functional networks in cognitively normal, mild cognitive impairment, and Alzheimer’s disease. PLoS One, 8(1), e53922. 10.1371/journal.pone.0053922, 23335980 PMC3545923

[bib74] Shine, J. M., & Poldrack, R. A. (2018). Principles of dynamic network reconfiguration across diverse brain states. NeuroImage, 180, 396–405. 10.1016/j.neuroimage.2017.08.010, 28782684

[bib75] Siliverstovs, B., & Herzer, D. (2007). Manufacturing exports, mining exports and growth: Cointegration and causality analysis for Chile (1960–2001). Applied Economics, 39(2), 153–167. 10.1080/00036840500427965

[bib76] Smoliński, Ł., & Członkowska, A. (2016). Cerebral vasomotor reactivity in neurodegenerative diseases. Neurologia i Neurochirurgia Polska, 50(6), 455–462. 10.1016/j.pjnns.2016.07.011, 27553189

[bib77] Song, J., Birn, R. M., Boly, M., Meier, T. B., Nair, V. A., Meyerand, M. E., & Prabhakaran, V. (2014). Age-related reorganizational changes in modularity and functional connectivity of human brain networks. Brain Connectivity, 4(9), 662–676. 10.1089/brain.2014.0286, 25183440 PMC4238253

[bib79] Trakoshis, S., Martínez-Cañada, P., Rocchi, F., Canella, C., You, W., Chakrabarti, B., … Lombardo, M. V. (2020). Intrinsic excitation-inhibition imbalance affects medial prefrontal cortex differently in autistic men versus women. eLife, 9, e55684. 10.7554/eLife.55684, 32746967 PMC7402681

[bib80] Tremblay, J. C., & Ainslie, P. N. (2021). Global and country-level estimates of human population at high altitude. Proceedings of the National Academy of Sciences, 118(18), e2102463118. 10.1073/pnas.2102463118, 33903258 PMC8106311

[bib81] van den Heuvel, M. P., de Lange, S. C., Zalesky, A., Seguin, C., Yeo, B. T. T., & Schmidt, R. (2017). Proportional thresholding in resting-state fMRI functional connectivity networks and consequences for patient-control connectome studies: Issues and recommendations. NeuroImage, 152, 437–449. 10.1016/j.neuroimage.2017.02.005, 28167349

[bib83] Vinck, M., Oostenveld, R., van Wingerden, M., Battaglia, F., & Pennartz, C. M. A. (2011). An improved index of phase-synchronization for electrophysiological data in the presence of volume-conduction, noise and sample-size bias. NeuroImage, 55(4), 1548–1565. 10.1016/j.neuroimage.2011.01.055, 21276857

[bib84] Virués-Ortega, J., Buela-Casal, G., Garrido, E., & Alcázar, B. (2004). Neuropsychological functioning associated with high-altitude exposure. Neuropsychology Review, 14(4), 197–224. 10.1007/s11065-004-8159-4, 15796116

[bib85] Vlisides, P. E., Bel-Bahar, T., Lee, U., Li, D., Kim, H., Janke, E., … Mashour, G. A. (2017). Neurophysiologic correlates of ketamine sedation and anesthesia: A high-density electroencephalography study in healthy volunteers. Anesthesiology, 127(1), 58–69. 10.1097/ALN.0000000000001671, 28486269 PMC5478453

[bib86] Voytek, B., & Knight, R. T. (2015). Dynamic network communication as a unifying neural basis for cognition, development, aging, and disease. Biological Psychiatry, 77(12), 1089–1097. 10.1016/j.biopsych.2015.04.016, 26005114 PMC4443259

[bib87] Voytek, B., Kramer, M. A., Case, J., Lepage, K. Q., Tempesta, Z. R., Knight, R. T., & Gazzaley, A. (2015). Age-related changes in 1/f neural electrophysiological noise. Journal of Neuroscience, 35(38), 13257–13265. 10.1523/JNEUROSCI.2332-14.2015, 26400953 PMC4579381

[bib88] Vyšata, O., Procházka, A., Mareš, J., Rusina, R., Pazdera, L., Vališ, M., & Kukal, J. (2014). Change in the characteristics of EEG color noise in Alzheimer’s disease. Clinical EEG and Neuroscience, 45(3), 147–151. 10.1177/1550059413491558, 24131619

[bib89] Wang, H. E., Bénar, C. G., Quilichini, P. P., Friston, K. J., Jirsa, V. K., & Bernard, C. (2014). A systematic framework for functional connectivity measures. Frontiers in Neuroscience, 8, 405. 10.3389/fnins.2014.00405, 25538556 PMC4260483

[bib90] Watts, D. J., & Strogatz, S. H. (1998). Collective dynamics of ‘small-world’ networks. Nature, 393(6684), 440–442. 10.1038/30918, 9623998

[bib91] Welch, P. (1967). The use of fast Fourier transform for the estimation of power spectra: A method based on time averaging over short, modified periodograms. IEEE Transactions on Audio and Electroacoustics, 15(2), 70–73. 10.1109/TAU.1967.1161901

[bib92] Wig, G. S. (2017). Segregated systems of human brain networks. Trends in Cognitive Sciences, 21(12), 981–996. 10.1016/j.tics.2017.09.006, 29100737

[bib93] Zhao, J.-P., Zhang, R., Yu, Q., & Zhang, J.-X. (2016). Characteristics of EEG activity during high altitude hypoxia and lowland reoxygenation. Brain Research, 1648, 243–249. 10.1016/j.brainres.2016.07.013, 27421182

